# Dual-channel fluorescent sensors based on chitosan-coated Mn-doped ZnS micromaterials to detect ampicillin

**DOI:** 10.1038/s41598-024-59772-3

**Published:** 2024-05-02

**Authors:** Son Hai Nguyen, Van-Nhat Nguyen, Mai Thi Tran

**Affiliations:** 1 School of Mechanical Engineering, Hanoi University of Science and Technology, Hanoi, 100000 Vietnam; 2 College of Engineering and Computer Science, VinUniversity, Hanoi, 100000 Vietnam; 3 VinUni-Illinois Smart Health Center, VinUniversity, Hanoi, 100000 Vietnam

**Keywords:** Biological techniques, Biotechnology, Optical techniques

## Abstract

The global threat of antibiotic resistance has increased the importance of the detection of antibiotics. Conventional methods to detect antibiotics are time-consuming and require expensive specialized equipment. Here, we present a simple and rapid biosensor for detecting ampicillin, a commonly used antibiotic. Our method is based on the fluorescent properties of chitosan-coated Mn-doped ZnS micromaterials combined with the β-lactamase enzyme. The biosensors exhibited the highest sensitivity in a linear working range of 13.1–72.2 pM with a limit of detection of 8.24 pM in deionized water. In addition, due to the biological specificity of β-lactamase, the proposed sensors have demonstrated high selectivity over penicillin, tetracycline, and glucose through the enhancing and quenching effects at wavelengths of 510 nm and 614 nm, respectively. These proposed sensors also showed promising results when tested in various matrices, including tap water, bottled water, and milk. Our work reports for the first time the cost-effective (Mn:ZnS)Chitosan micromaterial was used for ampicillin detection. The results will facilitate the monitoring of antibiotics in clinical and environmental contexts.

## Introduction

Detecting antibiotics is crucial for various reasons across multiple sectors. In medical diagnostics, specific antibiotics can indicate potential issues such as antibiotic resistance or overdosing, and they can also monitor patients’ adherence to their prescriptions^1^. Environmental testing of antibiotics is essential due to the risk of antibiotics entering the environment via agricultural runoff or improper medicine disposal, thereby contributing to the development of antibiotic-resistant bacteria^2^. In the agricultural industry, testing for antibiotics ensures food safety by checking that no residues remain in consumable products, which could otherwise contribute to antibiotic resistance in humans^3^. Specifically, its detection is significant for ampicillin (AMP) due to its common usage and the emergence of ampicillin-resistant bacteria, making it a key aspect of managing antibiotic resistance^4^.

Detecting low concentrations of ampicillin in complex matrices is challenging due to potential interference from other substances and the need for high sensitivity and specificity. Traditional methods employed for this purpose include High-Performance Liquid Chromatography (HPLC), Liquid Chromatography-Mass Spectrometry (LC–MS), Enzyme-Linked Immunosorbent Assay (ELISA), and Microbial Assays^5–7^. While these methods have proven effective, they often require sophisticated equipment, trained personnel, and sample pre-treatment. They may not always be suitable for real-time or on-site detection. To overcome the limitations of traditional antibiotic detection methods, researchers have turned their attention towards biosensors based on nanomaterials and micromaterials^8^. Biosensors have become an increasingly popular tool for detecting antibiotics such as AMP due to their specificity, sensitivity, and potential for rapid detection^9–11^. Different biosensors like electrochemical, optical, or field-effect transistor (FET)-based biosensors are developed with varying degrees of success^12–15^. For instance, electrochemical biosensors translate biochemical reactions into detectable changes in electrical properties, such as current, potential, or impedance. A field-effect transistor (FET)-based biosensor utilizes the modulation of electronic properties upon target molecule binding. On the other hand, optical biosensors detect shifts in optical properties such as fluorescence, absorbance, or refractive index stemming from antibiotic interactions. Among them, optical biosensors based on micromaterials have emerged as a prominent tool in antibiotic detection, combining the remarkable optical properties of micromaterials with the selectivity of biorecognition elements^16,17^.

Enzymes, known for their ability to confer resistance to antibiotics, have recently been harnessed as potent tools for detecting the presence of these antibiotics in various samples^18–20^. When integrated into detection assays or fluorescence biomarkers, the hydrolytic action of enzymes can signal the presence of antibiotics through observable changes, such as the color transformation seen with chromogenic substrates. Moreover, the specificity of certain enzyme variants allows for the differentiation of specific antibiotics in a group. However, distinguishing individual members in a group is still challenging for biosensors, especially fluorescent biosensors. To overcome this problem, biomarkers, serving as indicative biological molecules, can bring about discernible changes in the fluorescence signal when in contact with certain materials. This alteration might manifest as fluorescence quenching or enhancement, allowing for the sensitive and specific detection of the biomarker.

Among the array of fluorescent materials, (Mn:ZnS)CH emerges as a preferred choice as a fluorescence biomarker. This combination has become a key strategy in the continuous search to improve the sensitivity and specificity of antibiotic detection methods, establishing them as vital biomarkers^21,22^. ZnS, particularly when doped with Mn, exhibits excellent photoluminescence properties, which can be harnessed for sensitive and specific detection of target analytes, including antibiotics^23^. On the other hand, chitosan, a naturally derived polysaccharide, brings notable biocompatibility, non-toxicity, and a rich array of functional groups that can be utilized to immobilize specific bioreceptors^24^. Investigations into using chitosan-coated Mn-doped ZnS for antibiotic biosensor development are still in their early stages, and few studies have documented their use for this purpose. However, these preliminary studies suggest significant potential. For instance, one recent work reported the successful application of Mn-doped ZnS quantum dots in detecting chloramphenicol and tetracycline residues, demonstrating the high sensitivity and specificity of the biosensor in a complex matrix^23^. Despite these promising results, no specific studies have been conducted on detecting AMP using these chitosan-coated Mn-doped ZnS micromaterials. Therefore, our research endeavors to broaden the utility of chitosan-capped Mn-doped ZnS micromaterials for AMP detection, thereby addressing a substantial void in the existing literature. To the best of our understanding, this study represents the first documented analysis of an AMP optical biosensor utilizing chitosan-capped Mn-doped ZnS microparticles. This report will serve as a proof-of-concept for a sensor capable of detecting AMP in the range of 13.1–72.2 pM. In this study, we also present a sensing platform that can distinguish AMP from different selected analytes, including penicillin G (PCN), tetracycline (TET), and glucose. The working performance of proposed sensors based on (Mn:ZnS)CH to detect AMP using beta-lactamase in different matrices such as tap water, bottled water, and milk is also examined. Moreover, the stability of the proposed sensors over a month was explored. Our work aims to lay the foundation for a simple yet powerful biosensing platform capable of differentiating and detecting AMP with high sensitivity, stability, and selectivity.

## Results and discussion

### Characterizations of the synthesized material

**Figure 1:**
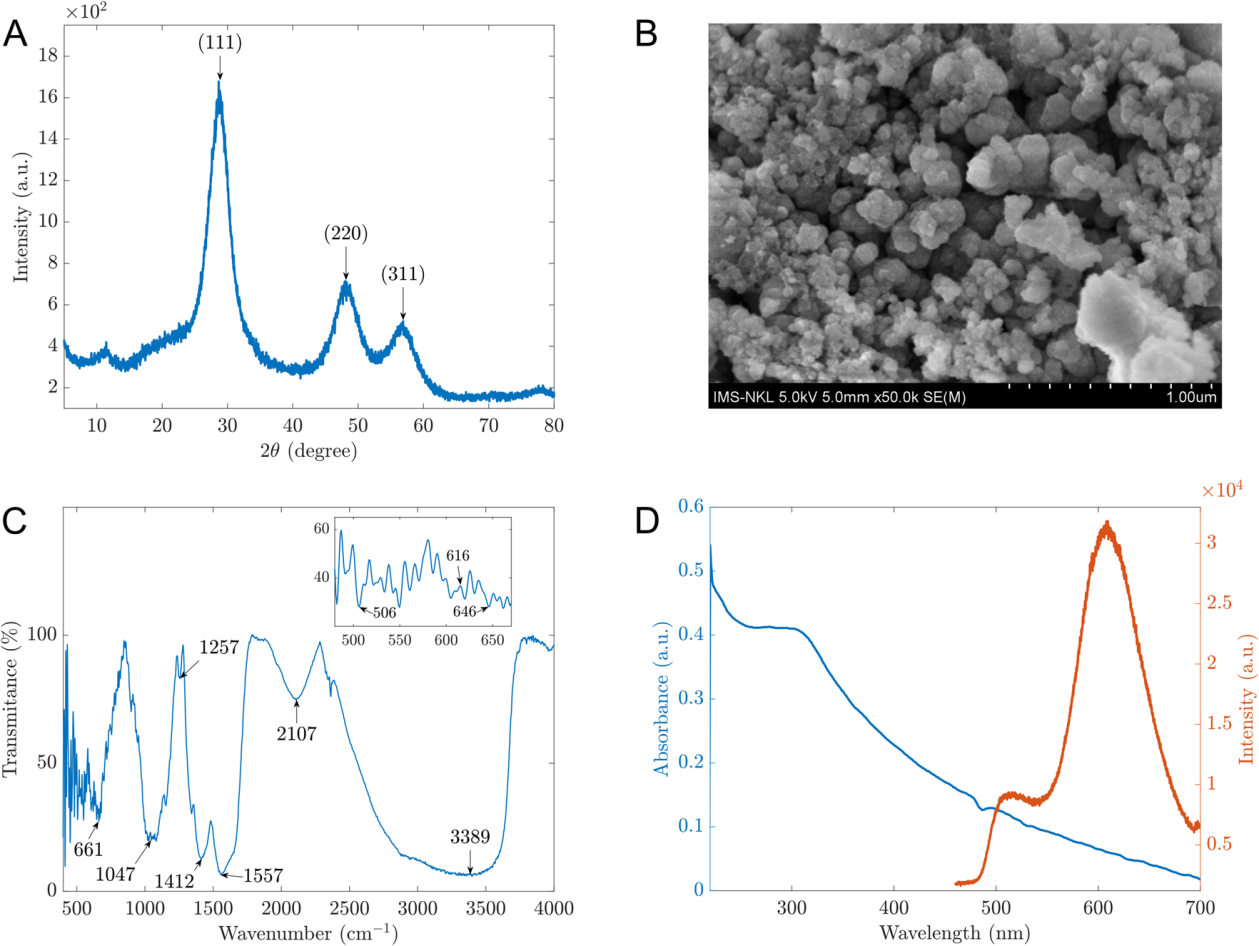
(**A**) XRD patterns; (**B**) SEM image; (**C**) FTIR spectra; (**D**) Absorbance (blue) and photoluminescent (orange) spectra of the synthesized material.

The synthesized materials were examined by the XRD and SEM images as the initial stage in characterizing their structure and shapes. As shown in Fig. 1A, three peaks at positions (111), (220), and (311) corresponding to the angle 2*θ* of 28.92^ο^, 48.44^ο^, and 57.26^ο^, respectively, demonstrated the cubic sphalerite structure of ZnS (JCPDS Card No. 5-0566). This observation indicates that ZnS materials were synthesized successfully, Mn^2+^ and chitosan were also observed in XRD patterns with some small peaks at (221), (310), and (431). However, they may not significantly impact the structure of ZnS due to a low percentage. Furthermore, the microparticles of the prepared material can be displayed by the SEM image in Fig. 1B with sizes of about 0.2 to 0.5 µm. In Fig. 1C, the FTIR analysis was performed with the FT/IR-4600 Jasco spectrometer (Jasco, Japan) across the 400–4000 cm^−1^ spectrum. The chitosan-coated ZnS doped with Mn exhibited a distinctive peak at 3389 cm^−1^, indicative of the N–H bend in the primary amine group (–NH_2_), confirming the presence of chitosan. Chitosan's spectral signature also includes peaks at 1412 cm^−1^ and 1557 cm^−1^ for the –CO bond, at 1047 cm^−1^ for the –C–O–C bond. Specific vibrational peaks of ZnS were identified at 1047, 616, and 508 cm^−1^. A notable splitting of the peak at 1047 cm^−1^ into two distinct peaks suggests that the Mn doping altered the structural integrity of some ZnS particles. Additionally, a peak at 661 cm^−1^ is attributed to Mn^25–29^. Before utilizing (Mn:ZnS)CH for AMP detection, the optical properties of the synthesized samples were examined. Figure 1D depicts the absorption and photoluminescent (PL) spectra of (Mn:ZnS)CH micromaterials. The PL peaks are observed at 510 nm and 614 nm when excited with 365 nm wavelength.

### Fluorescence biosensors based on (Mn:ZnS)CH micromaterials to directly detect Ampicillin treated with β-lactamase

**Figure 2:**
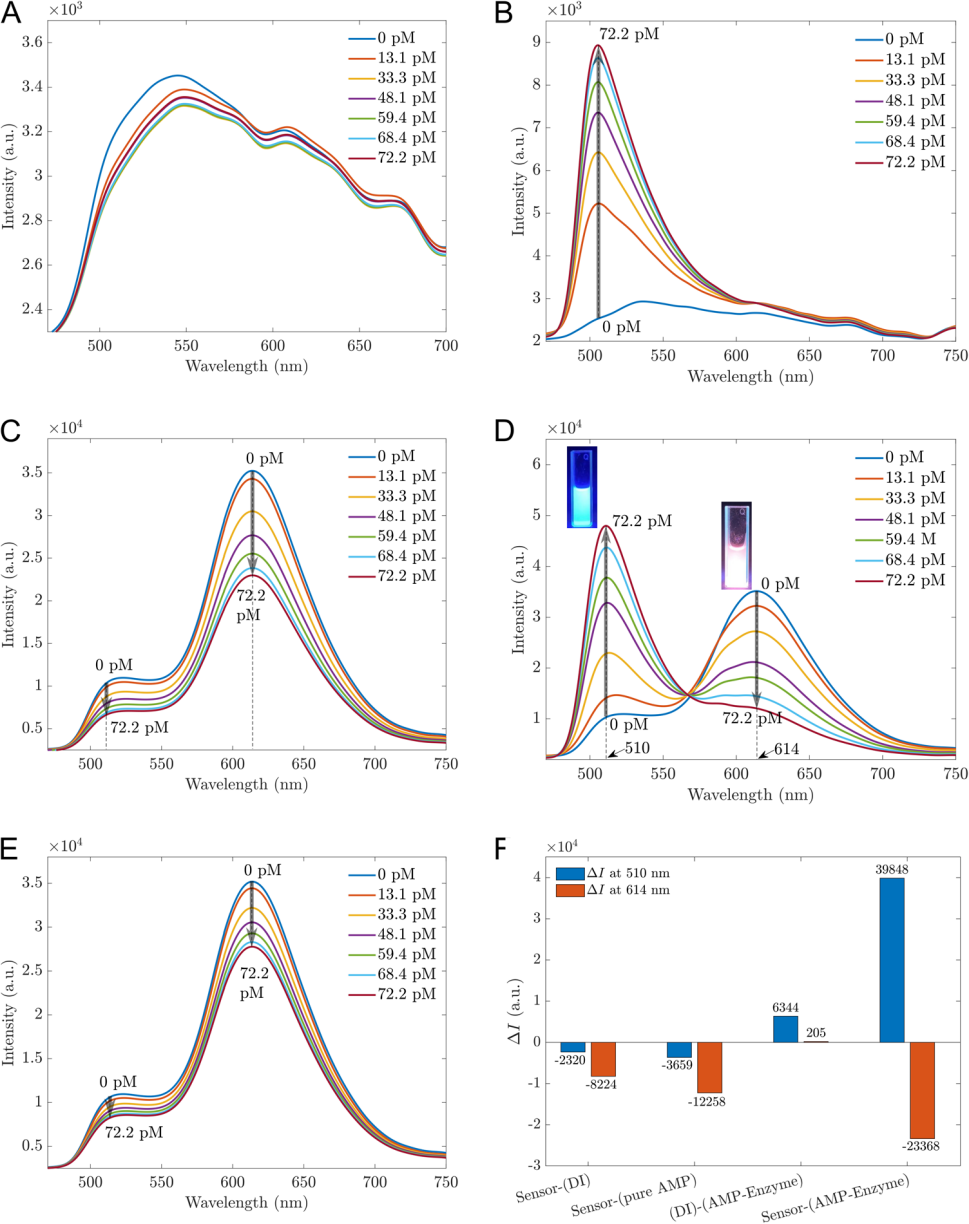
Fluorescent spectra of different pure AMP concentrations (**A**) and AMP-Enzyme (**B**); Fluorescent sensors based on (Mn:ZnS)CH micromaterials with pure AMP (**C**) and with AMP-Enzyme (**D**). The legends are the concentrations of pure AMP and AMP-Enzyme in (**A**, **C**) and (**B**, **D**), respectively; (**E**) Biosensors based on (Mn:ZnS)CH nanomaterials with DI water, the legends represent for needed DI water volume added equivalent to the antibiotics concentrations; (**F**) The differences in intensity $\Delta I$ at 510 nm and 614 nm before and after adding 72.2 pM.

Fluorescent sensors have garnered significant attention due to their ability to detect small change signals^30,31^. In the context of AMP biosensors, the enzyme β-lactamase plays a crucial role as a bio-recognition element due to its unique interaction with β-lactam antibiotics such as AMP. β-lactamase catalyzes the hydrolysis of the β-lactam ring, a core structural component of AMP, resulting in a significant change in the antibiotic’s molecular structure. This enzymatic reaction can be exploited to generate a measurable signal in biosensors. In this study, taking advantage of the properties of β-lactamase, pure AMP was treated by this enzyme as described in the Method section. Then the sensors based on (Mn:ZnS)CH materials can indirectly detect AMP via byproducts of AMP treated by β-lactamase (AMP-Enzyme). In our experiment, the working performance of the proposed sensors was evaluated with AMP concentration range of 13.1–72.2 pM. First, the photoluminescent intensity of pure AMP is depicted in Fig. 2A, showing small changes across different AMP concentrations. However, the enzymatic oxidation occurs when ampicillin interacts with β-lactamase enzyme at the suggested pH of 7.4, resulting in byproducts with fluorescent properties, as shown in Fig. 2B. As the concentration of AMP varied from 13.1 to 72.2 pM, a single fluorescence peak at 510 nm was observed, with the fluorescent intensity increasing from 5200 to 8900. These fluorescent byproducts are considered a pointer compound to indirectly determine the concentration of AMP. When the byproducts were in contact with sensors based on (Mn:ZnS)CH, the fluorescence at the 510 nm peak was enhanced significantly from 10^4^ to 4.7 × 10^4^, while the 614 nm peak was gradually quenched with increasing AMP concentrations (Fig. 2D). This enhancing effect at 510 nm contrasts to the case of pure AMP in Fig. 2C and that of added deionized (DI) water to the sensor in Fig. 2E.

To visualize better the enhancing and quenching effects for each experiment, the intensity differences ($\Delta I)$ at 510 nm and 614 nm before and after adding 72.2 pM are illustrated in Fig. 2F. The result confirmed an interaction between byproducts and the surface of (Mn:ZnS)CH, which enhanced the fluorescence at 510 nm. The change in this dual-channel can quickly identify the effect of AMP-Enzyme with the significant changes at two peaks of 510 nm and 614 nm.

**Figure 3:**
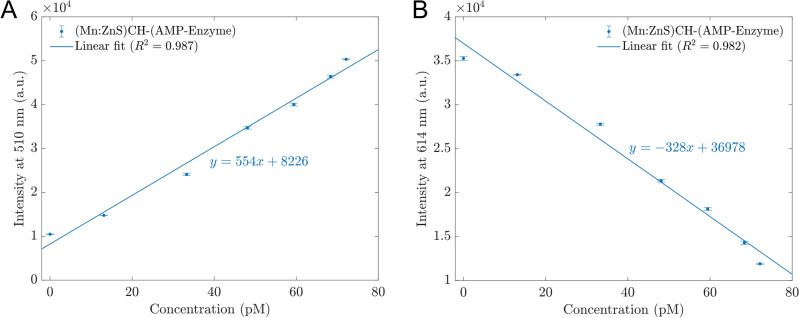
The photoluminescence intensities at the wavelength 510 nm (**A**) and 614 nm (**B**) vary with the concentrations of analytes. The error bars represent the standard deviations of nine measurements.

Figure 3A,B illustrate the linear relationship between the AMP-Enzyme concentration and the intensity of the sensor at 510 nm and 614 nm, respectively. Based on the slopes of the linear fits, it is recommended the calibration line at 510 nm for quantitative measurement:

1 $$y = \, 554x + \, 8226$$

where *y* is the photoluminescence intensity at 510 nm, *x* is the concentration of AMP-enzyme (pM). This sensor has a detection limit of 8.24 pM using the S/N = 3 method, which is Limit of Detection (LoD) = Blank signal + 3 standard derivations.

To validate our method, we prepared three samples with AMP-Enzyme concentrations of 41.23, 54.11, and 64.13 pM. We recorded the PL spectra of these samples when they interacted with the sensor and then used the intensities at 510 nm to determine the AMP-Enzyme concentrations using Eq. (1). The results are presented in Table 1. The estimated concentrations of the test samples were in good agreement with the actual concentrations, with all discrepancies being smaller than 10%.

**Table 1:** Validation of the proposed sensors.

Test concentration (pM)	Intensity at 510 nm (a.u.)	Estimated concentration (pM)	Difference (%)
41.23	29,388.67	38.20	7.35
54.11	37,349.56	52.57	2.85
64.13	42,656.44	62.15	3.09

**Figure 4:**
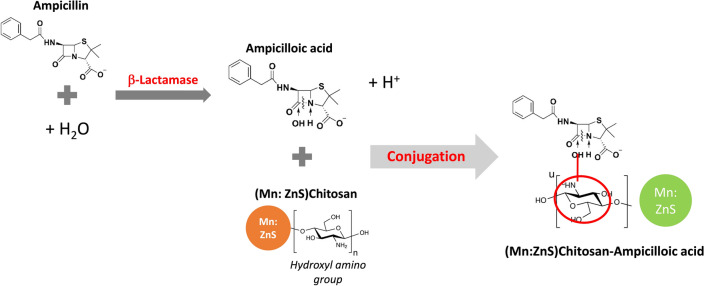
The schematic to illustrate the working mechanism of PL detection of proposed sensors.

**Figure 5:**
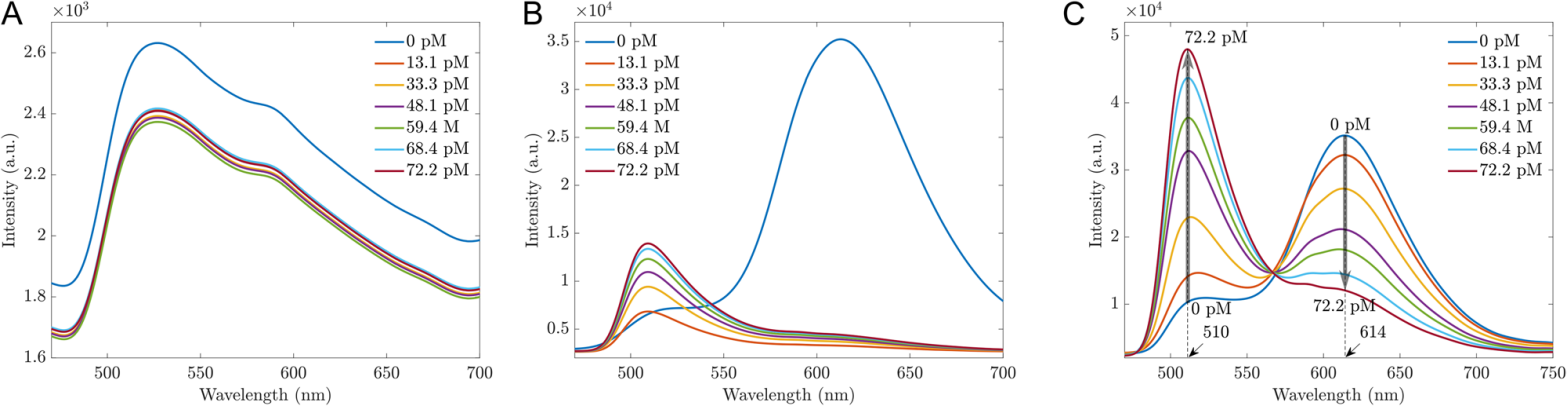
The PL spectra of the sensors were based on different sensing materials in contact with AMP-Enzyme; the sensing materials were (**A**) ZnS; (**B**) Mn:ZnS; (**C**) (Mn:ZnS)CH.

The working mechanism can be explained as shown in Fig. 4. The enzyme used in this work can destroy β ring of β-lactam antibiotics and inactivate the antibiotics. The enzyme was encouraged to be used in an environment with a pH of 7.4 and a temperature of 37 °C. After the breakage of the amide bond, AMP would degrade and form ampicilloic acid and H^+^ in the water solution^32^. The hydrolyzed byproducts were produced and in contact with (Mn:ZnS)CH nanoparticles. The hydroxyl amino groups in chitosan might be mainly responsible for their various properties, such as reducing activity, which leads to the formation of nanoparticles, or their ability to bond with byproducts of the reactions between AMP and β-lactamase enzyme via electrostatic forces, hydrogen bonding or covalent interactions^10,33,34^. After the conjugation between ampicilloic acid and (Mn:ZnS)CH nanoparticles, the emission peak enhanced at 510 nm when excited by 365 nm wavelength. The fluorescence increases with an increasing amount of ampicilloic acid conjugated with (Mn:ZnS)CH, confirming the conjugation. An increase in ampicillin concentration in the conjugate was observed to be proportional to the increase in fluorescence, as shown in Fig. 2D. To validate this hypothesis, we prepared different materials with the same hydrothermal conditions. The resultant materials including Mn:ZnS and ZnS were characterized in Supplement 1. As shown in Fig. 5, the PL spectra of these materials (blue lines) differed significantly, particularly in the case of ZnS, which had no Mn doping and exhibited a single peak at approximately 520 nm. The presence of chitosan did not alter the PL spectra. However, their behaviors varied significantly when the sensors, based on different sensing materials, came into contact with AMP-Enzyme. When the sensing material was ZnS, the photoluminescence was weak, around 2600, and slightly decreased upon the addition of AMP-Enzyme (Fig. 5A). In the case of Mn:ZnS (Fig. 5B), the sensor exhibited a high peak at 614 nm, potentially due to Mn-doped atoms. However, when AMP-Enzyme was added, the 614 nm peak was completely quenched, and the intensity at 510 nm increased from 0.7 × 10^4^ to 1.5 × 10^4^ (doubling when the concentration of 72.2 pM AMP-Enzyme was added). On the other hand, in Fig. 5C, when chitosan was coated on Mn:ZnS, the peak at 614 nm was gradually quenched, and the intensity at 510 enhanced significantly from 10^4^ to 5 × 10^4^ (five times with the higher initial intensity). Clearly, chitosan played a key role and was responsible for improving the PL intensity and sensitivity of proposed sensors, especially the enhancing effect at 510 nm even though their pH values were close to 6.06, 6.63, and 6.24 for (Mn:ZnS)CH, (Mn:ZnS), and ZnS, respectively.

### Selectivity and stability of proposed sensors

To examine the selectivity of the proposed sensor, under the same conditions, the prepared sensor was used to detect pure AMP, AMP-Enzyme, pure PCN, PCN-Enzyme, pure TET, TET-Enzyme, Glucose, and Glucose-Enzyme and compared the intensities at 510 nm and 614 nm wavelength. We intended to test the selectivity of the proposed sensors with one beta-lactam antibiotic (PCN), one antibiotic without a beta-lactam ring (TET), and one analyte that is not an antibiotic (glucose). Because PCN and AMP are β-lactam antibiotics, the enzyme used in this work can destroy β ring of β-lactam antibiotics and inactivate both antibiotics. The proposed sensors work at room temperature and have a pH of 6.06. The enzyme was mixed with AMP, PCN, TET, and glucose in DI, and the pH of different analytes was then measured. The results showed that the pH of pure AMP, AMP-Enzyme, pure PCN, PCN-Enzyme, pure TET, TET-Enzyme, glucose, and glucose-Enzyme were 7.48, 7.97, 5.06, 6.63, 2.53, 2.89, 3.78, and 6.11, respectively. After adding analytes into the proposed sensors, only the case of AMP-Enzyme has a pH of 7. The fluorescence spectra of different cases are recorded and shown in Supplement 2. Only in the case of AMP, the PL spectra were significantly different between pure and treated enzyme samples. Two other antibiotics and glucose have similar patterns in the two experiments. For TET and TET-Enzyme, the highest fluorescence peak shifted (from 614 to 593 nm) when we added antibiotics. Glucose only induced the quenching effect, similar to Glucose-Enzyme, PCN pure, and PCN- Enzyme. Based on the PL spectra, AMP-Enzyme can be differentiated. It seems that at pH conditions of 7 and room temperature, only the beta-lactam ring of AMP was destroyed, and the byproducts combined with the sensors’ surface produced the signal changes.

**Figure 6:**
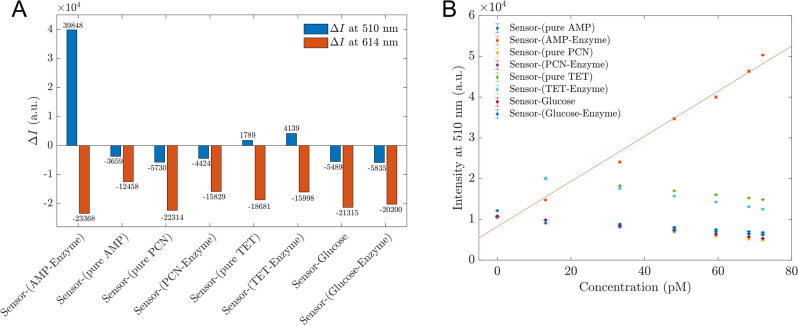
(**A**) The intensity changes in dual channel of 510 nm and 614 nm for proposed sensors detecting different analytes; (**B**) The photoluminescent intensity at 510 nm of proposed sensors in contact with different analytes changed with the concentrations of analytes. These intensities are obtained from Supplement 2.

To visualize these differences better and more quantitatively, the intensity difference before and after 72.2 pM analytes at dual channels were demonstrated in Fig. 6A, and the intensities at 510 nm when sensors were in contact with different analytes were established as shown in Fig. 6B. Figure 6A demonstrates significant changes at both 510 nm and 614 nm of AMP-Enzyme, with distinct patterns that enable differentiation from other analytes. Furthermore, Fig. 6B indicates that only samples of AMP treated with enzyme produced a positive slope, reflecting an enhancing effect and substantial changes in intensity at 510 nm. In contrast, other analytes exhibited negative slopes within the 13.1–72.2 pM range, corresponding to quenching effects.

**Figure 7:**
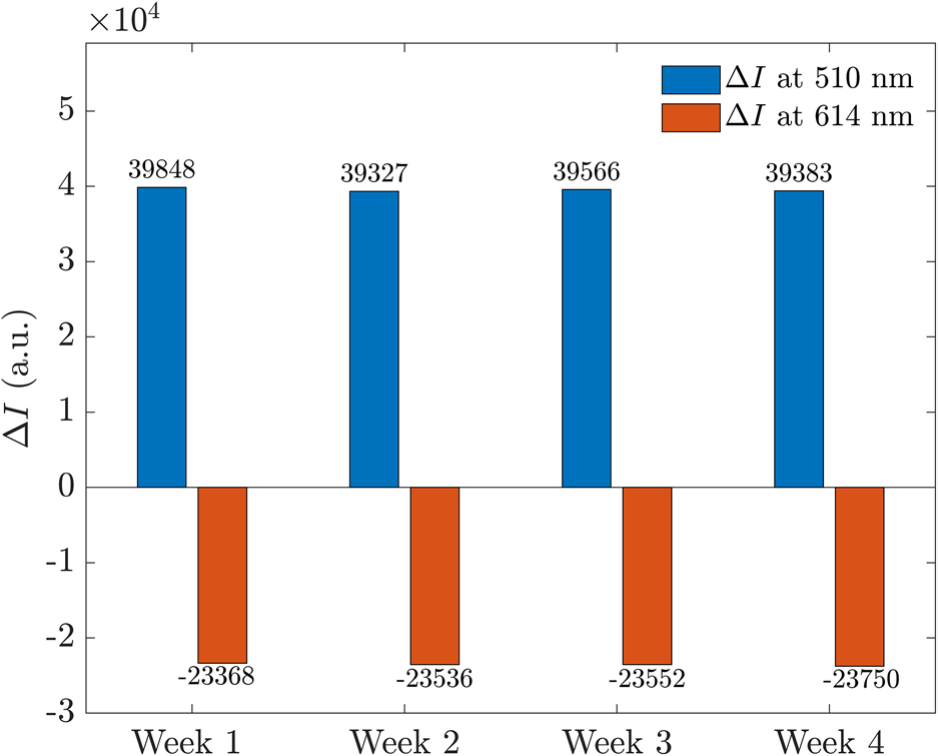
The stability of the proposed sensors is demonstrated by the intensity changes of proposed sensors in contact with AMP-Enzyme at dual channels of wavelength 510 nm and 614 nm over 4 weeks.

Next, we investigated the stability of the proposed sensors by repeating the PL measurements of proposed sensors in contact with AMP- Enzyme weekly over a period of four weeks (the photoluminescence spectra was shown in Supplement 3). The intensity changes at 510 nm and 614 nm were determined and displayed in Fig. 7. The stable results confirm the high stability of our proposed sensors in response to AMP-Enzyme over time.

### The potential of the proposed sensors working in different matrices

**Figure 8:**
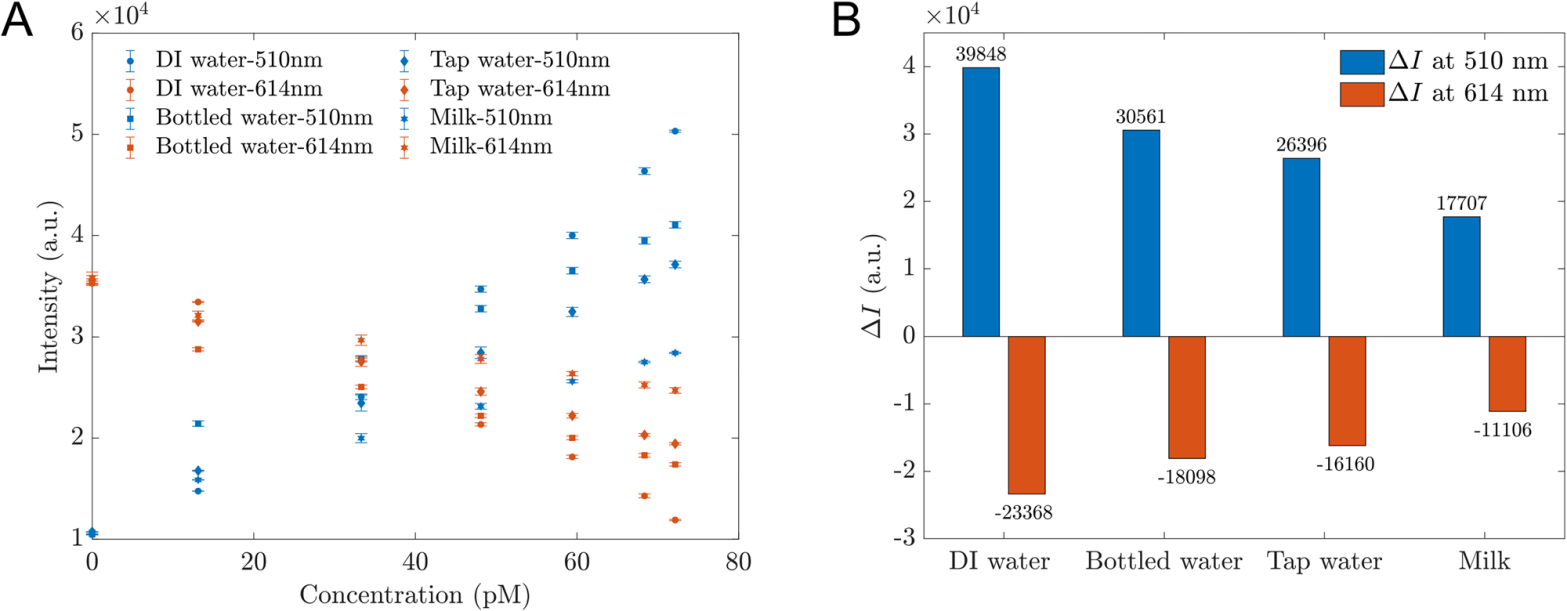
(**A**) The intensities of the proposed sensors in different matrices including DI, tap water, bottled water, and milk at wavelengths of 510 nm and 614 nm. The error bar represents the standard deviation of 9 measurements; (**B**) The intensity changes of proposed sensors in contact with AMP-Enzyme at dual channels of wavelength 510 nm and 614 nm when detecting AMP-Enzyme in DI water, bottled, tap water, and milk.

As the initial step in developing sensors that function in practice, we expanded our experiments to study the proposed sensors in various matrices. These included tap water supplied by the Hanoi Water Limited Company, bottled water and organic milk without any antibiotics or sugar. Due to its cream color, which absorbs and emits a small amount of light, the milk was diluted 30 times with DI water before any measurements were taken (this diluted milk is referred to as ‘milk’ for simplicity). The PL spectra of the proposed sensors with AMP-Enzyme in these different matrices showed identical shapes and trends. The intensities at 510 nm and 610 nm are shown in Fig. 8A. Figure 8B depicts the differences in intensity before and after adding 72.2 pM AMP-Enzyme. The enhancing and quenching effects were the highest when the proposed sensors were used in DI water, with gradually lower sensitivity (lower slope and smaller changes) observed when used in bottled water, tap water and milk, respectively. In all cases, the intensity at 510 changed linearly as a function of AMP-Enzyme concentration, as shown in Table 2. However, the LOD of sensors working in tap water was the smallest (3.01 pM). These results showed the high potential of the proposed sensors working in real reality.

**Table 2:** Working functions and LODs of the proposed sensors when working in DI water, tap water, bottled water, and milk.

Matrix	Working function	R^2^	LOD (pM)
DI water	*y* = 554*x* + 8226	0.987	8.24
Bottled water	*y* = 389.62*x* + 13,550	0.972	12.25
Tap water	*y* = 356.69*x* + 11,378	0.998	3.01
Milk	*y* = 233.38*x* + 11,794	0.990	7.22

*x* is the AMP concentration (pM), *y* is the intensity at 510 nm.

This study introduces, for the first time, a simple and cost-effective sensor based on (Mn:ZnS)CH to detect AMP with high sensitivity, selectivity, and stability. While there have been some reports on AMP sensors, these alternatives either possess higher LODs or require more complex and costly preparation processes or different detection techniques. For instance, O.I. Guliy and colleagues described a sensor for AMP detection using a microwave electrodynamic resonator, wherein a porous polystyrene film was utilized to immobilize long-living Escherichia coli K-12 cells. This sensor could detect AMP levels ranging from 4 to 50 μg/ml with an LOD of 4 μg/ml^35^. Rahul Kumar Mishra et al. introduced optical sensors based on carbon dots, capable of detecting AMP concentrations from 0.165 × 10^–4^ to 4.957 × 10^–4^ M, with an LOD of 0.165 × 10^–4^ M^36^. Omid Heydaru and Raouf Ghavami developed absorbance sensors using gold nanoparticles, which could identify AMP in the range of 1 to 600 nM with a LOD of 0.1 nM^37^. Xiaohong Shi and associates presented electrochemical sensors based on Electropolymerized Poly(o-Phenylenediamine)/Gold Nanoparticle/Single-Walled Carbon Nanotube, detecting AMP from 5.0 × 10^–8^ to 1.0 × 10^–5^ mol/L with an LOD of 1 nM^38^.

## Conclusions

This study has successfully demonstrated the feasibility of using cost-effective and environmentally friendly chitosan-coated Mn-doped ZnS nanomaterials as sensing materials for detecting AMP. AMP, pretreated by β-lactamase enzyme, was detected with high sensitivity and selectivity by a simple fluorescent sensor. The proposed sensors based on (Mn: ZnS)CH nanomaterials proved highly accurate, stable, and sensitive in detecting AMP linearly in the range 13.1–72.2 pM in various matrices, achieving the highest sensitivity with the detection limit of 8.24 pM for fluorescence sensor working in DI water, which is unprecedented for a simple optical biosensor. Overall, with the ability to accurately detect trace amounts of AMP, this technology has the potential to significantly impact various fields, including healthcare, environmental monitoring, and food safety.

## Methods

### Chemicals

In this work, we used the chemicals without any further purification as follows: Zinc acetate dihydrate Zn(CH_3_COO)_2_·2H_2_O (Merck, Germany), Sodium sulfide nonahydrate Na_2_S·9H_2_O (98%, China), Manganese chloride tetrahydrate MnCl_2_·4H_2_O (Merck, Germany), Chitosan (C_6_H_11_NO_4_) (90%, Shanghai Zhanyun Chemical Co., Ltd, Shanghai, China), Ethanol C_2_H_5_OH (99.5%, Xilong Scientific Co., Ltd., Guangdong, China), β-lactamase (C_21_H_17_N_3_O_8_S_3_, CAS # 9073-60-3, purity ≥ 95%, Chins, D-Glucose monohydrate (99.99%, C_6_H_12_O_6_.H_2_O, Biobasic, Canada), Penicillin G sodium salt (ultra- pure, C_16_H_17_N_2_NaO_4_S, Bomeibio, China); Ampicillin sodium salt C_16_H_18_N_3_NaO_4_S (99.99%, Njduly, China), Tetracycline hydrochloride C_22_H_24_N_2_O_8_. HCl (TET, ultra-pure, Biobasic, Ontario, Canada), and distilled water.

### Preparation of chitosan-coated Mn-doped ZnS nanomaterials

The chitosan-coated Mn-doped ZnS material was prepared by the hydrothermal method, adhering to the protocol outlined in the previous reports^22,39^ and illustrated in Supplement 4. Initially, we dissolved 1.5% of chitosan in 1% acetic acid. Then, Zn(CH_3_COO)_2_ and MnCl_2_ were combined in a mole ratio of 10:1, stirring the mixture until it dissolved completely. Subsequently, we gradually added a suspension of 0.5 g Na_2_S and 1% wt chitosan to this mixture, stirring it for an hour. After this period, we transferred the suspension to a Teflon container and maintained it at 80 °C for two hours. The resultant precipitation was washed thrice with ethanol and centrifuged at 5000 rpm for four minutes. Finally, the product was dried at 60 °C for seven hours.

### Antibiotics treatment

Antibiotics were mixed with the β-lactamase enzyme in a 1:1 ratio and diluted to the desired concentration before the experiments. These mixtures were stored at 3 °C. The pH of all samples was checked before use.

### Fluorescence setups and measurements

In our experimental setup, we introduced 1500 µL of sensing materials, specifically (Mn:ZnS)CH, at a concentration of 2500 mg/L into cuvettes with a thickness of 10 mm. Subsequently, we incrementally added 100 µL of AMP treated by beta-lactamase to the cuvette to achieve various concentration levels ranging from 13.1 to 72.2 pM. We then recorded the fluorescence intensities using a spectrophotometer (SpectraPro HRS-300, Teledyne Princeton Instruments, Trenton, NJ 08619 USA) with a 10 nm slit-width. This process was done at an excitation wavelength of 365 nm and an exposure time of just 1 s.

### Supplementary Information

Supplementary Figures.

## Data Availability

The datasets used and analyzed during the current study are available from the corresponding author upon reasonable request.
