# Supplementary material for: Dual-channel fluorescent sensors based on chitosan-coated Mn-doped ZnS micromaterials to detect ampicillin
Source: Sci Rep. 2024 May 2;14:10066. doi: 10.1038/s41598-024-59772-3 (PMC11065863; doi:10.1038/s41598-024-59772-3)
Supplement: Supplementary file 1 — Supplementary Figures. [file 41598_2024_59772_MOESM1_ESM.docx]

**Dual-channel fluorescent sensors based on Chitosan-coated Mn-doped ZnS micromaterials to detect Ampicillin**

**Authors:** Son Hai Nguyen^1^, Van-Nhat Nguyen^2^, Mai Thi Tran^2,3*^

**Affiliations:**

^1^School of Mechanical Engineering, Hanoi University of Science and Technology, Hanoi 100000, Vietnam

^2^College of Engineering and Computer Science, VinUniversity, Hanoi 100000, Vietnam

^3^VinUni-Illinois Smart Health Center, VinUniversity, Hanoi 100000, Vietnam

*Corresponding author: mai.tt@vinuni.edu.vn

**Supplement 1.** The SEM image, XRD pattern, and FTIR spectra of: (A, C, E) ZnS nanorods; (B, D, F) Mn:ZnS nanoparticles.


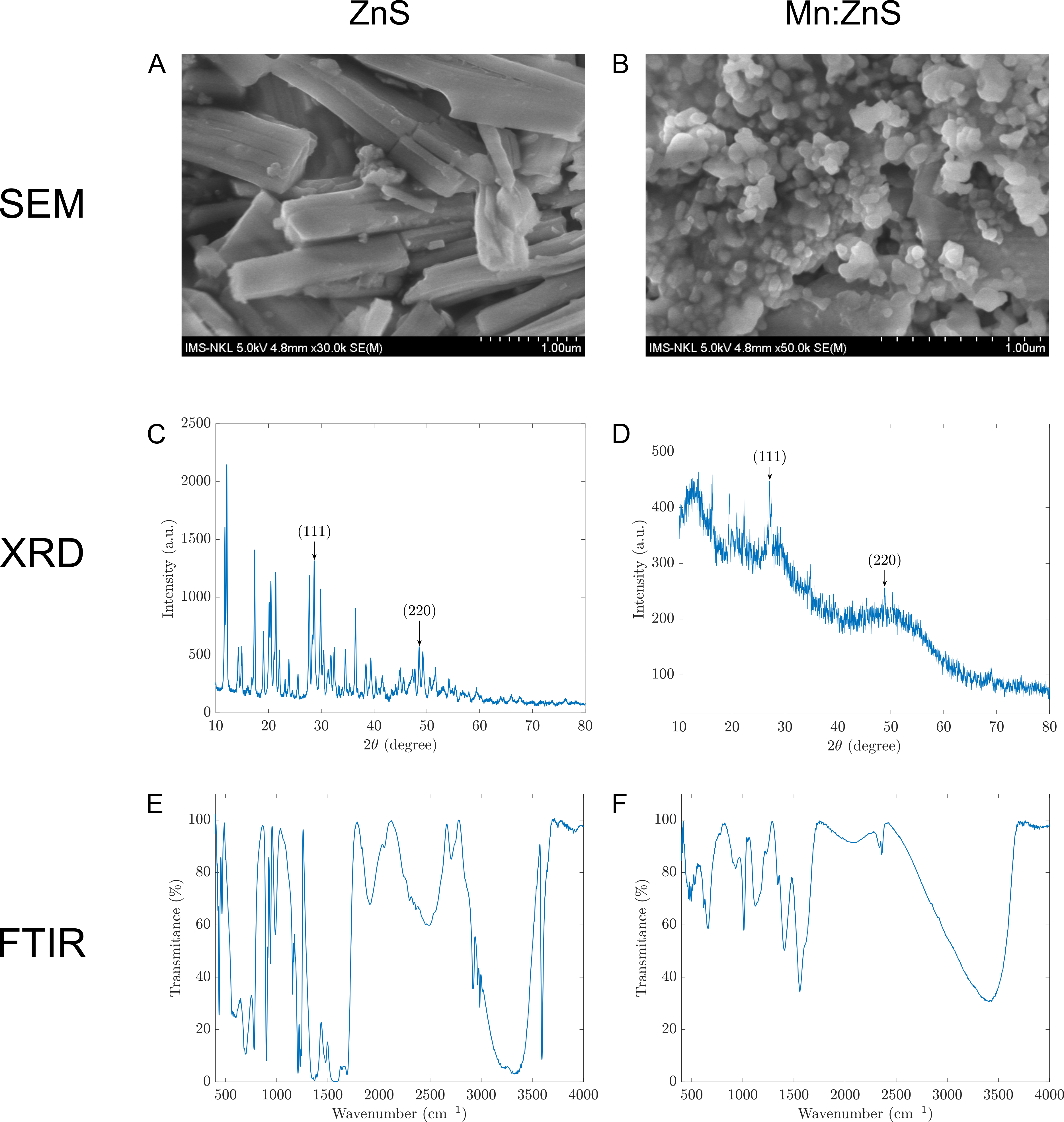


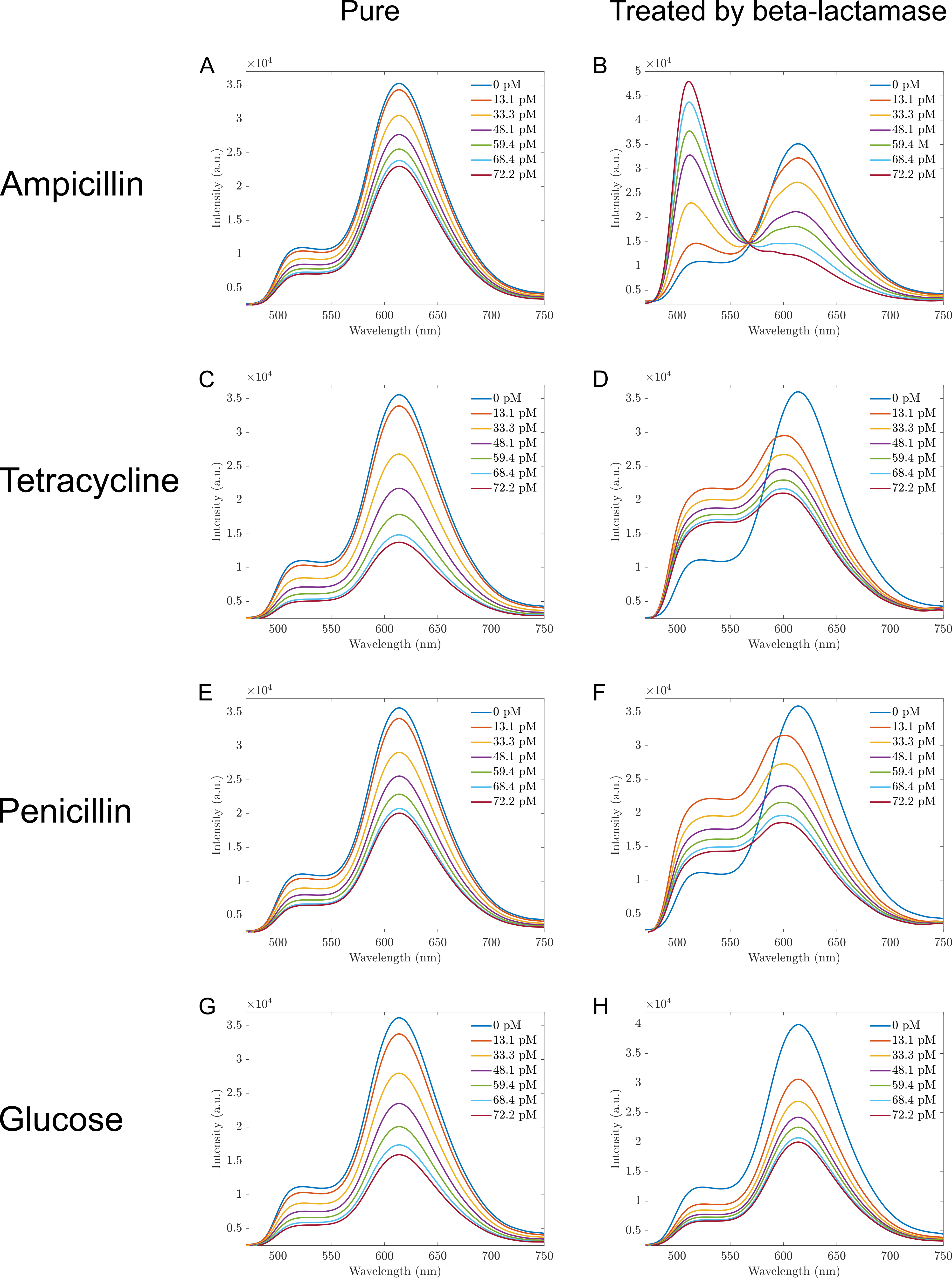


**Supplement 2.** Photoluminescence of proposed sensors with: (A) pure AMP, (B) AMP-Enzyme, (C) pure PCN, (D) PCN-Enzyme, (E) pure TET, (F) TET-Enzyme, (G) Glucose, (H) Glucose-Enzyme.


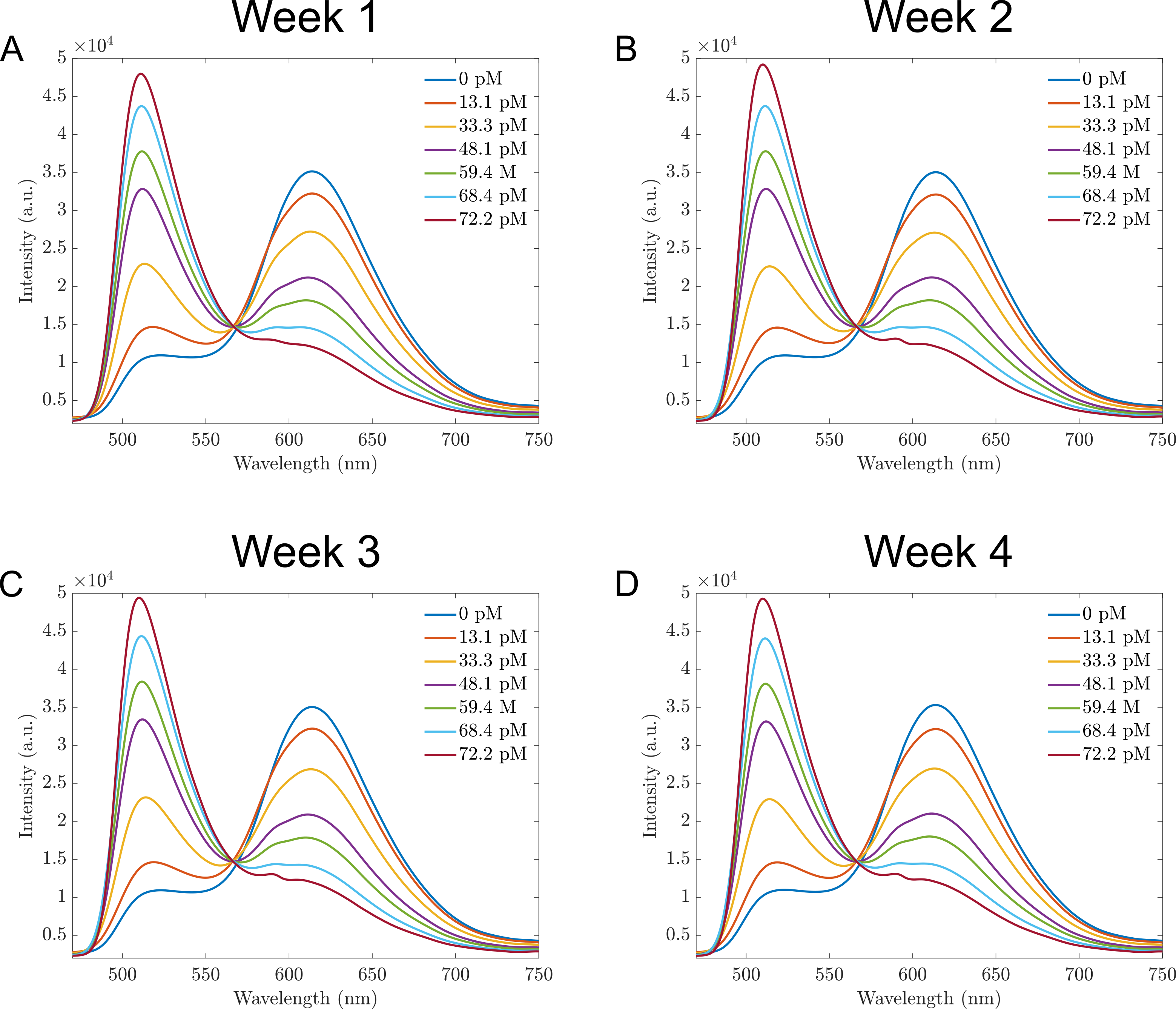


**Supplement 3.** The fluorescence spectra of proposed sensors in contact with AMP- Enzyme for 4 weeks. The sensors and analytes were kept for four weeks to test the stability.


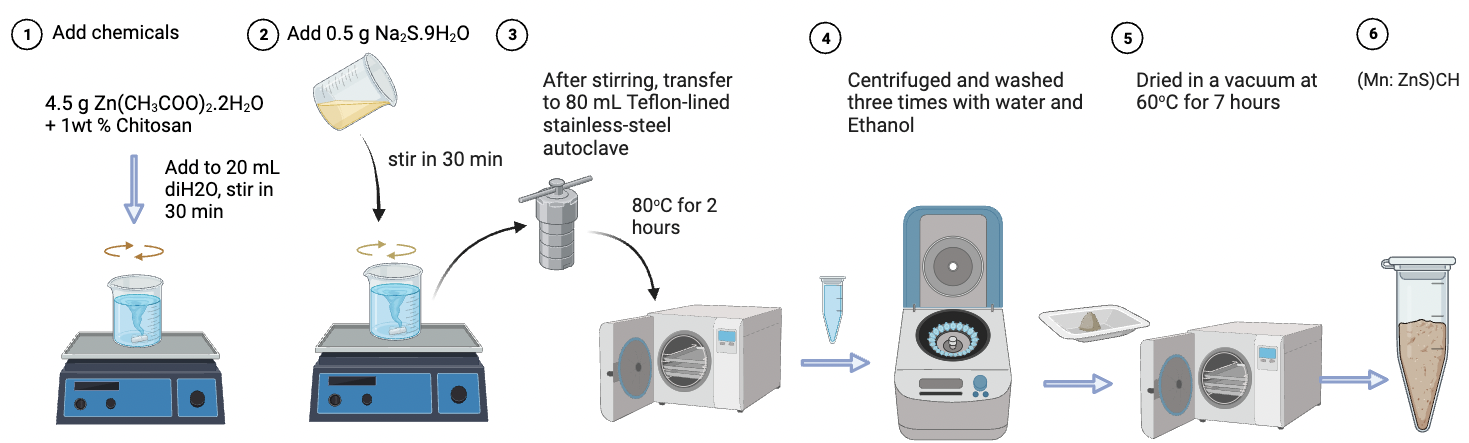


**Supplement 4.** The schematic of an experimental procedure to prepare ZnS-doped Mn-capped chitosan nanomaterials by hydrothermal method.
